# B cell and anti-PLA2R antibody-guided rituximab therapy in idiopathic membranous nephropathy: a prospective multi-center cohort study in the East Coastal Region of China

**DOI:** 10.3389/fimmu.2025.1633532

**Published:** 2025-09-17

**Authors:** Yili Xu, Liang Wang, Chunming Jiang, Dong Sun, Min Yang, Jin Liu, Xiaobin Liu, Cheng Wan, Caixia Liu, Bo Zhang, Guangyu Bi, Lianhua Chen, Liyuan Zhang, Guoyuan Lu, Liang Zhang, Hua Zhou, Xiaobo Zhang, Gang Zhou, Fang Lu, Chengning Zhang, Bin Sun, Ming Zeng, Shuaibo Bian, Li Zhang, Ningning Wang, Lei Shen, Yanggang Yuan, Changying Xing, Huijuan Mao

**Affiliations:** ^1^ Department of Nephrology, the First Affiliated Hospital of Nanjing Medical University, Jiangsu Province Hospital, Nanjing, China; ^2^ Department of Nephrology, The Affiliated Wuxi People’s Hospital of Nanjing Medical University, Wuxi, China; ^3^ Department of Nephrology, Nanjing Drum Tower Hospital, the Affiliated Hospital of Nanjing University Medical School, Nanjing, China; ^4^ Department of Nephrology, the Affiliated Hospital of Xuzhou Medical University, Xuzhou, China; ^5^ Department of Nephrology, The First People’s Hospital of Chang Zhou City, Changzhou, China; ^6^ Clinical Medicine Research Institution, The First Affiliated Hospital of Nanjing Medical University, Jiangsu Province Hospital, Nanjing, China; ^7^ Department of Nephrology, Northern Jiangsu People’s Hospital, Affiliated Hospital to Yangzhou University, Yangzhou, China; ^8^ Department of Nephrology, The Affiliated Huai’an No.1 People’s Hospital of Nanjing Medical University, Huai’an, China; ^9^ Department of Nephrology, The first people’s hospital of Lianyungang, Lianyungang, China; ^10^ Department of Nephrology, The First Affiliated Hospital of Soochow University, Suzhou, China; ^11^ Department of Nephrology, The Affiliated Suqian First People’s Hospital of Nanjing Medical University, Suqian, China

**Keywords:** idiopathic membranous nephropathy, rituximab, targets driven, prospective cohort, non-randomized clinical trials

## Abstract

**Introduction:**

This study assessed the safety and efficacy of B cell- and anti-PLA2R antibody-targeted low-dose rituximab therapy in patients with idiopathic membranous nephropathy (IMN).

**Methods:**

This was a multicenter, investigator-initiated, open-label, prospective cohort study. Patients were recruited from 10 hospitals in the east coastal region of China between November 1^st^, 2019 and June 15^th^, 2023. Enrolled patients were assigned to individualized rituximab therapy (guided by peripheral B cells and anti-PLA2R antibody levels) or standard rituximab therapy (1,000 mg × 2 or 375 mg/m² × 3–4): the individualized group (n = 78) and the standard group (n = 62). Odds ratios (ORs) and 95% confidence intervals (CIs) for response were estimated using multivariate logistic regression models, adjusting for key confounders, with inverse probability of treatment weighting (IPTW) applied to balance demographic and clinical characteristics. The primary outcome was a composite of complete or partial remission of proteinuria.

**Results:**

A total of 140 patients were included in the sta tistical analysis, which was completed on June 10^th^, 2024. After IPTW, baseline characteristics were well balanced between the two groups. Patients were followed every 2 months for 1 year after the first rituximab injection. At 12 months, 57 of 78 patients (73.1%) in the individualized therapy group and 40 of 62 patients (64.5%) in the standard therapy group achieved complete or partial remission [the adjusted risk difference and 95% CI were 0.1 (–0.05 to 0.26); p = 0.001 for noninferiority]. In the weighted cohort, 74.1% in the individualized group and 70.5% in the standard group achieved remission (p = 0.5). The median (interquartile range) total rituximab dose per patient at 1 year was 800 mg (600–1,100 mg), with a total cost of RMB 16,227.5 (13,148–23,536) per unit utility in the individualized group, which was markedly lower than in the standard group. Anti-PLA2R autoantibody negativity at 6 months post-treatment predicted a higher probability of remission. The frequency of adverse events differed significantly between groups (6.4% vs. 12.9%, *P* = 0.02).

**Discussion:**

B cell- and anti-PLA2R antibody-targeted rituximab therapy may be a cost-effective and safe alternative for patients with IMN. Randomized controlled trials with larger samples are needed to confirm these findings.

**Clinical Trial Registration:**

https://www.chictr.org.cn/showproj.html?proj=42793, identifier ChiCTR1900026382.

## Introduction

Idiopathic membranous nephropathy (IMN) is a common cause of nephrotic syndrome in adults (1). Persistent moderate to severe proteinuria is an independent risk factor for end-stage kidney disease (ESKD), leading to higher medical costs, prolonged hospitalization, and more complications (2). Standard rituximab treatment strategies recommended by various guidelines for patients with IMN include four weekly doses of 375 mg/m^2^ (3–5) or two doses of 1,000 mg on days 1 and 15 (6). However, dosing schedules vary worldwide, and results remain inconsistent (7–9). The high cost of rituximab is also a major concern, particularly in resource-limited settings.

Recently, a monthly mini-dose regimen guided by CD20^+^ B cell counts and anti-PLA2R titers was recommended based on a population pharmacokinetic/pharmacodynamic model (10). This model was initially developed in 41 patients with primary membranous nephropathy (PMN) using a quantitative dose–exposure–response relationship through a mechanistic target-mediated drug disposition (TMDD) model, followed by regression analysis of anti-PLA2R titer reduction over time after treatment.

In light of these considerations, we conducted a multicenter, nonrandomized, concurrent controlled trial across 10 hospitals in eastern China. We designed an individualized treatment protocol titrated to circulating B cells and anti-PLA2R antibody levels and compared its outcomes with those of a standard regimen (375 mg/m^2^ weekly for 4 weeks or 1,000 mg on days 0 and 15). The aim was to investigate whether individualized therapy would be noninferior to standard therapy over 12 months in patients with IMN.

## Methods

### Trial design and oversight

This investigator-initiated, open-label, multicenter, prospective cohort study was conducted at 10 sites in coastal cities of Jiangsu Province, eastern China. The study design has been reported previously (11). The study was conducted and reported in accordance with the STROBE checklist.

### Participants

Beginning November 1^st^ 2019, patients presenting with nephrotic syndrome at the 10 participating nephrology centers were screened for eligibility. Inclusion criteria were as follows: (1) biopsy-proven membranous nephropathy (MN) at first diagnosis with moderate or high risk of renal progression, or refractory MN; (2) age 18–75 years with proteinuria >3.5 g per 24 h and serum albumin <30 g/L; and (3) CD19^+^ B lymphocyte count >5/mm³. Exclusion criteria were: (1) estimated glomerular filtration rate (eGFR) <30 mL/min/1.73 m²; (2) infant or childhood-onset nephrotic syndrome; (3) secondary MN; (4) abnormal liver function (greater than two times the upper limit of normal); (5) pregnancy or breastfeeding; (6) active infectious diseases, such as chronic hepatitis B, hepatitis C, AIDS, or tuberculosis; (7) severe impaired immune response, such as hypoimmunoglobulinemia (IgG <4 mg/dL), CD4 cell count <200/mm³, or CD19^+^ B lymphocyte count < 5/mm^3^, (8) major cardiovascular or cerebrovascular events (e.g., myocardial infarction, heart failure, cerebral hemorrhage) within the past 6 months; (9) systemic immunosuppressant use for more than 2 weeks within 12 weeks before screening, with inability to discontinue or taper. Immunosuppressants (cyclophosphamide, mycophenolate mofetil, azathioprine, or *Tripterygium* wilfordii) were prohibited for at least 3 months before enrollment. For refractory MN previously treated with calcineurin inhibitors (CNIs), tacrolimus or cyclosporine could be continued at tapering doses with serum concentrations maintained at 4–8 ng/mL (tacrolimus) or 100–150 ng/mL (cyclosporine) at study entry. Steroid doses did not exceed 20 mg/day. (10) Allergy to rituximab or any excipient in the formulation. All patients were followed for 12 months after hospital discharge.

The study was approved by the ethics committee of the First Affiliated Hospital of Nanjing Medical University (2019-SR-452.A1) and the ethics committees of the other 9 sites. The trial was registered in the Chinese Clinical Trial Registry (ClinicalTrials.gov identifier ChiCTR1900026382). Informed consent was obtained for treatment, follow-up, and tissue and blood sampling.

### Definitions

Refractory membranous nephropathy (12, 13) was defined as the absence of clinical and/or immunological remission (i.e., antibody titer below the detection threshold by ELISA or a negative indirect immunofluorescence assay) after a course of treatment with corticosteroids and a calcineurin inhibitor (CNI).

Moderate or high risk of renal progression was defined according to KDIGO guidelines: Moderate risk: Normal eGFR; proteinuria > 3.5g/d and not decreased >50% after 6 months of conservative therapy with an ACEi/ARB; and not fulfilling high-risk criteria. High risk: eGFR <60 ml/min/1.73 m² and/or proteinuria >8g/d for >6 months; or normal eGFR, proteinuria >3.5 g/day and not decreased by >50% after 6 months of conservative therapy with ACEi/ARB, plus at least one of the following: serum albumin <25g/l, anti-PLA2R antibody >50 RU/mL, urinary IgG>1ug/min, urinary α1-microglobulin >40 µg/min, urinary β2-microglobulin >250 mg/day, or selectivity index >0.20 (14).

### Procedures

In view of personal preference, patients were assigned into these two groups: individualized therapy and standardized therapy according to their willingness.

### Individualized therapy: Low-dose rituximab (B cell- and anti-PLA2R-targeted)

Strategy of individualized low-dose (B cell- and anti-PLA2R-targeted) therapy was conducted according to the treatment schedule of IMN patients assigned in our previous study (11). The initial dose of rituximab depended on the body surface area (BSA), and the minimum dose was 150 mg/m² BSA. Considering that the half-life of rituximab (RTX) is approximately 3 weeks (15), the second injection was scheduled 2 weeks later to maintain stable therapeutic drug concentrations. After that, follow-up was conducted at an interval of 2 months. At each follow-up, if >5 B cells per mm³ were observed, (1) if the PLA2R antibody decreased or was negative, 100 mg was administered; (2) if the PLA2R antibody was unchanged, 75 mg/m^2^ was given; (3) if the PLA2R antibody was higher than before, 150 mg/m² was given. If <5 B cells per mm³ were observed, (1) if the PLA2R antibody decreased or was negative, no additional course; (2) if the PLA2R antibody was unchanged, 100 mg was administered; (3) if the PLA2R antibody was higher than before, 75 mg/m² was given.

In brief, our initial dose during the first month was determined by peripheral B cells and body surface area. Follow-up every 2 months thereafter was planned. The subsequent dosage depended on the anti-PLA2R antibody titer level and B cell count.

#### Standardized therapy

In total, 1,000 mg on days 0 and 15 or 375 mg/m² rituximab weekly for 3 to 4 weeks were received. Then, laboratory indexes were measured every 2 months. If the anti-PLA2R antibody persisted at the 6th month (9 patients), rituximab was administered in a lower dosage of RTX (150mg/m^2^).

Inclusion in either group was based on the patient’s preference.

### Concomitant therapy

All patients received optimal supportive care, including renin–angiotensin system blockers and blood pressure management. For refractory MN, the doses of calcineurin inhibitors could be maintained or tapered with serum trough concentrations within 4–8 ng/mL (tacrolimus) or 100–150 ng/mL (cyclosporine) after entering the study. Steroids did not exceed 20 mg daily. Patients were followed for 12 months after discharge from the hospital.

### Sample size

Sixty-three patients per group would provide 80% power to detect noninferiority regarding complete remission (CR) or partial remission (PR) at 12 months at a one-sided significance level of 0.025 (equivalent to a two-sided significance level of 0.05) and a noninferiority margin of 15 percentage points on an absolute risk difference scale, assuming that 55% of patients in the individualized rituximab group and 45% of those in the standard therapy group achieved CR or PR at 12 months. Considering a 10% dropout rate, at least 70 patients per group were enrolled.

### Outcome measurement

The primary clinical outcome was the composite of complete or partial remission at 12 months. Secondary outcomes were time to remission, progression to end-stage kidney disease (ESKD), anti-PLA2R levels, proteinuria, estimated glomerular filtration rate, and adverse events.

The following definitions were used: complete or partial remission, proteinuria <0.3 g/24 h or <3.5g/24h and <50% of baseline, respectively, in at least two consecutive visits; relapse, recurrence of massive proteinuria >3.5 g/24 h and serum albumin <30g/L on two of three consecutive days; CD19^+^ B cell depletion, CD19^+^ B cell count <5/mm^3^. For safety evaluation, immediate infusion reactions (within 48 h) were recorded. Serious infections were defined as any infection requiring hospitalization and/or intravenous antibiotics or resulting in disability or death.

### Statistical analyses

The analysis was performed using R software version 4.2. Continuous variables with normal distribution were expressed as mean ± standard deviation and analyzed using Student’s t-test. Nonnormally distributed continuous variables were expressed as median (interquartile range, IQR) and tested using the Wilcoxon rank-sum test. Categorical variables were presented as n (%), and the chi-squared test or Fisher’s exact test was used. Logistic regression was used to calculate risk difference (RD) and 95% confidence interval (CI) to compare remission rates between individualized therapy and standard therapy. Survival rates were estimated by Kaplan–Meier analysis, and survival curves were compared using the log-rank test.

We applied inverse probability of treatment weighting (IPTW) by computing stabilized weights inversely proportional to the probability of treatment assignment to control for potential confounders. Standardized differences were used to assess balance in characteristics between groups after IPTW, with an absolute value <0.10 considered negligible. The missing indicator method was applied for covariates with missing data. Covariates included MN clinical type, laboratory values, and medications. Sociodemographic characteristics at index date (age, sex, BMI, systolic/diastolic blood pressure, and duration of proteinuria), prior therapies, and therapies during follow-up were also included. A p-value <0.05 was considered statistically significant.

## Results

### Participants

**Figure 1:**
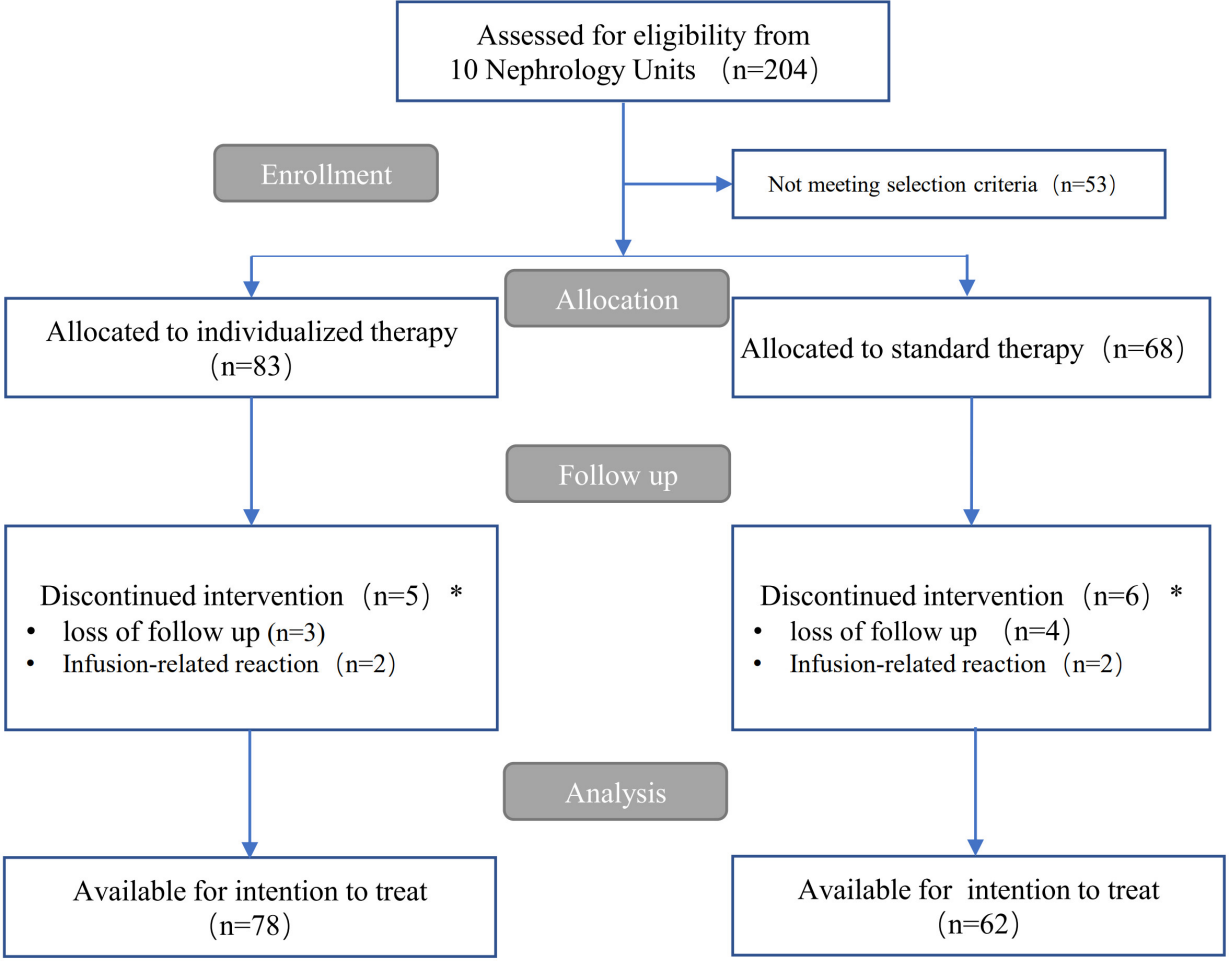
Flow chart of patients who received individualized therapy and standard therapy.

Among 204 patients referred to our 10 nephrology units from 1 November 2019 to 15 June 2023, 53 did not meet the inclusion criteria. The remaining 151 patients were assigned to each rituximab therapy group for the management of IMN. Five participants in the individualized group and six in the standard therapy group were excluded from statistical analysis because of loss to follow-up within 1 month after rituximab use, or due to infusion reactions at first administration leading to withdrawal after enrollment. Thus, they were not included in the overall effect evaluation. Over a median (interquartile range) follow-up of 12 (12, 16) months, a total of 140 patients (78 in the individualized therapy group and 62 in the standard therapy group) were available for statistical analysis (see flow chart in **Figure 1**).

The baseline parameters of the two groups are shown in **Table 1**. The median age was 51 years (SD, 14.59) in the individualized group and 52 years (SD, 13.99) in the standard group (standardized difference, 0.059); 51.2% and 50.8% were men (standardized difference, 0.002), respectively; and 19% and 40% were initial rituximab users (standardized difference, 0.884), respectively (**Table 1**). Baseline characteristics were similar between the individualized and standard groups; however, most patients (64.5%) in the standard therapy group received rituximab as initial therapy, according to clinical criteria for assessing the risk of progressive loss of kidney function in the KDIGO (14) guidelines (14). Gender, age, BMI, eGFR, serum albumin, urinary protein, anti-PLA2R, CD19^+^ B cells, and IgG were well balanced between groups before IPTW. Differences in previous immunosuppressant use (P <0.001), immunosuppressant therapy during follow-up (P <0.001), and duration of proteinuria before rituximab initiation (P = 0.016) in the standard therapy group were further balanced after IPTW (P >0.05) (**Table 1**).

**Table 1:** Baseline characteristics for all enrolled patients before and after weighting.

		Before inverse probability of treatment weighting				After inverse probability of treatment weighting			
		Individual therapy	Standard therapy	p	Standardized difference	Individual therapy	Standard therapy	p	Standardized difference
n		78	62			107. 4	186. 4		
Gender (%)	male	48 (61. 5)	40 (64. 5)	0. 852	0. 062	60. 5 (56. 3)	77. 4 (41. 5)	0. 391	0. 3
	female	30 (38. 5)	22 (35. 5)			46. 9 (43. 7)	109. 0 (58. 5)		
Age (yr) [mean (SD)]		51. 92 (14. 59)	51. 95 (13. 99)	0. 991	0. 002	52. 24 (14. 34)	56. 00 (9. 93)	0. 116	0. 305
BMI (kg/m^2^) [mean (SD)]		25. 20 (4. 44)	24. 72 (3. 89)	0. 529	0. 117	25. 12 (4. 36)	25. 88 (3. 22)	0. 425	0. 199
Previous duration of proteinuria (mo) [mean (SD)]		32. 14 (50. 23)	14. 92 (23. 63)	0. 016	0. 439	28. 54 (45. 08)	29. 95 (29. 59)	0. 903	0. 037
systolic BP (mmHg) [mean (SD)]		128. 81 (16. 73)	130. 87 (18. 50)	0. 496	0. 117	128. 78 (16. 20)	127. 63 (19. 30)	0. 861	0. 065
diastolic BP (mmHg) [mean (SD)]		81. 21 (11. 55)	83. 63 (11. 01)	0. 215	0. 214	82. 26 (11. 47)	80. 34 (10. 56)	0. 592	0. 174
Clinical types of MN	Initial use, n (%)	19 (24. 4)	40 (64. 5)	<0. 001	0. 884	31. 5 (29. 3)	76. 0 (40. 8)	0. 487	0. 242
	RMN, n (%)	59 (75. 6)	22 (35. 5)			75. 9 (70. 7)	110. 4 (59. 2)		
Previous therapies before inclusion (%)	no medication, n (%)	19 (24. 4)	40 (64. 5)	<0. 001	0. 884	31. 5 (29. 3)	76. 0 (40. 8)	0. 487	0. 242
	steroids alone, n (%)	5 (6. 4)	3 (4. 8)			5. 8 (5. 4)	3. 9 (2. 1)		
	steroids and Calcineurin inhibitors, n (%)	54 (69. 2)	20 (32. 3)			70. 2 (65. 3)	138. 7 (74. 4)		
Therapy during follow-up (%)	no medication, n (%)	16 (20. 8)	52 (83. 9)	<0. 001	1. 713	37. 0 (34. 8)	60. 3 (32. 3)	0. 486	0. 324
	steroids alone, n (%)	42 (54. 5)	3 (4. 8)			45. 4 (42. 6)	103. 9 (55. 7)		
	steroids and Calcineurin inhibitors, n (%)	19 (24. 7)	7 (11. 3)			24. 0 (22. 6)	22. 2 (11. 9)		
eGFR (ml/min/1. 73 m²) [mean (SD)]		85. 09 (35. 52)	88. 39 (29. 43)	0. 558	0. 101	85. 42 (34. 89)	63. 96 (32. 18)	0. 031	0. 639
Serum albumin (g/L) [mean (SD)]		23. 40 (5. 41)	22. 49 (5. 42)	0. 328	0. 167	23. 58 (5. 33)	24. 08 (6. 58)	0. 841	0. 083
Urinary protein (g/d) [mean (SD)]		8. 60 (4. 83)	8. 02 (5. 17)	0. 496	0. 116	8. 21 (4. 71)	7. 64 (3. 83)	0. 568	0. 133
anti-PLA2R (RU/ml) [mean (SD)]		227. 38 (362. 29)	154. 49 (186. 59)	0. 175	0. 253	204. 79 (333. 30)	232. 28 (205. 75)	0. 718	0. 099
CD19 (/mm^3^) [mean (SD)]		282. 77 (197. 96)	315. 40 (234. 06)	0. 408	0. 151	312. 86 (212. 11)	328. 12 (143. 95)	0. 715	0. 084
IgG (mg/dL) [mean (SD)]		4. 89 (2. 01)	5. 67 (2. 77)	0. 103	0. 319	5. 25 (1. 98)	5. 07 (1. 81)	0. 705	0. 1

BMI, body mass index; eGFR, estimated glomerular filtration rate; Scr, serum creatine; CR, complete remission; PR, partial remission, RMN, refractory membranous nephropathy.

Data were presented as the mean ± standard, the median with interquartile range or counts and percentages. A two-tailed p<0. 05 was considered statistically significant.

### Treatment responses during follow-up

Primary outcome: remission at one year.

At 12 months of follow-up, 57 of 78 (73.1%) patients in the individualized therapy group and 40 of 62 (64.5%) in the standard therapy group achieved complete or partial remission (P <0.001). At 12 months, a significantly higher proportion of patients in individualized therapy (n=27; 34.6%) reached complete remission compared with standard therapy (n=11; 17.7%) (P <0.001) (**Table 2**). The median (IQR) remission time in both groups was 6 (4, 12) months. Multivariate logistic regression was used to evaluate treatment effect with adjustment for potential confounding variables. The outcome described whether patients maintained PR or CR within 12 months. Therapy strategy was included in the model along with other covariates, including gender, age, BMI, first-line rituximab use, anti-PLA2R titer, 24-h urinary protein, and use of steroids or immunosuppressants. Stepwise regression screening was performed, with both inclusion and exclusion criteria set at 0.05. The final model covariates were gender, 24-h urinary protein, and first-line rituximab use. The adjusted risk difference of response and 95% confidence interval was 0.1 (–0.05 to 0.26); the lower end of the confidence interval was above –15 percentage points, and the one-sided p-value for noninferiority was 0.001, meeting the significance threshold of α = 0.025 (**Table 3**). In unweighted cohorts, 73.1% of 78 individualized treatment patients and 66.1% of 62 standard treatment patients reached partial or complete remission (weighted risk ratio, 1.07 [95% CI, 0.92–1.25], p = 0.4). The weighted remission rates were 74.1% in the individualized group and 70.5% in the standard group (weighted risk ratio, 1.2 [95% CI, 0.71–2.07], p = 0.5) (**Table 4**).

**Table 2:** Follow-up information and efficacy outcomes variables before weighting.

Parameter	Total (n=140)	Individual therapy (n = 78)	Standard therapy (n = 62)	*P* value
Respondence, n (%)				
6 mo	76 (54. 3)	43 (55. 1)	33 (53. 2)	0. 138
12 mo	97 (69. 3)	57 (73. 1)	40 (64. 5)	< 0. 001
CR, n (%)				
6 mo	14 (10. 0)	11 (14. 1)	3 (4. 8)	< 0. 001
12 mo	38 (27. 1)	27 (34. 6)	11 (17. 7)	< 0. 001
PR, n (%)				
6 mo	62 (44. 3)	32 (41. 0)	30 (48. 4)	0. 002
12 mo	59 (42. 2)	30 (38. 5)	29 (46. 8)	0. 083
Urinary protein (g/d)				
Baseline	6. 79 (4. 59, 10. 56)	7. 33 (4. 73, 10. 69)	6. 13 (4. 44, 9. 54)	0. 533
6 mo	2. 57 (1. 08, 5. 97)	2. 24 (1. 15, 5. 65)	3. 18 (1. 05, 6. 12)	0. 996
12 mo	1. 08 (0. 28, 3. 74)	0. 95 (0. 23, 3. 68)	1. 36 (0. 4, 3. 9)	0. 768
eGFR (ml/min/1. 73 m²), median (IQR)				
Baseline	94. 96 (63. 79, 109. 61)	94. 63 (54. 91, 110. 71)	95. 05 (65. 10, 108. 25)	0. 695
6 mo	91. 78 (68. 71, 109. 38)	90. 52 (66. 09, 112. 24)	92. 50 (73. 58, 105. 44)	0. 491
12 mo	93. 95 (72. 57, 109. 22)	95. 88 (74. 25, 109. 60)	91. 62 (70. 09, 108. 35)	0. 719
Scr (μmol/L), median (IQR)				
Baseline	75. 20 (57. 90, 105. 00)	77. 00 (59. 60, 117. 35)	73. 50 (56. 75, 98. 25)	0. 106
6 mo	73. 20 (58. 00, 98. 95)	73. 90 (62. 13, 95. 48)	73. 00 (56. 00, 100. 50)	0. 788
12 mo	74. 00(60. 70, 102. 68)	74. 00(62. 00, 102. 40)	74. 00(57. 00, 105. 00	0. 782
Serum albumin (g/L), median (IQR)				
Baseline	22. 90 (19. 10, 26. 70)	22. 90 (20. 60, 27. 25)	22. 75 (18. 15, 26. 15)	0. 177
6 mo	33. 00 (25. 83, 38. 20)	32. 60 (24. 80, 37. 65)	34. 00 (26. 88, 38. 98)	0. 345
12 mo	38. 50 (33. 65, 41. 55)	38. 90 (34. 30, 42. 10)	38. 30 (32. 05, 41. 05)	0. 957
anti-PLA2R (RU/ml), median (IQR)				
Baseline	90. 93 (24. 20, 258. 81)	98. 58 (22. 70, 266. 21)	85. 00 (30. 94, 223. 00)	0. 201
6 mo	2. 00 (1. 53, 20. 75)	2. 07 (1. 43, 21. 05)	2. 00 (2. 00, 9. 32)	0. 073
12 mo	1. 77 (1. 39, 5. 49)	1. 63 (1. 40, 8. 7)	2. 00 (1. 34, 2. 42)	0. 091
CD19 (/mm^3^), median (IQR)				
Baseline	250. 62 (173. 44, 362. 32)	235. 52 (168. 10, 332. 27)	288. 00 (188. 00, 367. 00)	0. 517
6 mo	6. 01 (1. 90, 18. 58)	6. 76 (2. 76, 16. 34)	4. 20 (0. 00, 28. 93)	0. 107
12 mo	9. 40 (2. 26, 48. 75)	6. 42 (2. 24, 23. 10)	16. 10 (5. 23, 100. 00)	0. 024
Total dose of RTX (mg), median (IQR)				
6 mo	1000 (600, 2000)	700 (500, 900)	2000 (2000, 2400)	< 0. 001
12 mo	1300 (700, 2000)	800 (600, 1100)	2000 (2000, 2400)	< 0. 001
Cost (RMB, yuan), median (IQR)				
12 mo	27543. 6 (15803, 33477. 6)	16227. 5 (13148, 23536)	31265 (31265, 40163. 2)	< 0. 001

mo, months; eGFR, estimated glomerular filtration rate; Scr, serum creatine; CR, complete remission; PR, partial remission. Data were shown as the median with interquartile range or counts and percentages. A two-tailed p<0. 05 was considered statistically significant.

**Table 3:** The outcome of complete or partial remission.

Therapy	Event. No (%)	Crude risk difference	Adjust risk	Adjust risk difference	P value for noninferiority
Individualized therapy	22 (35)	0. 10 (-0. 05 to 0. 24)	0. 76	0. 10(-0. 05 to 0. 26)	0. 001
Standard therapy	20 (26)	0 (reference)	0. 66	0 (reference)	

Adjusted for gender, 24-hour urinary protein level and whether RTX was Initial use.

**Table 4:** Outcomes for remission in individualized group and standard therapy in the unweighted and Weighted Cohorts.

	Strategy	No. of patients	Remission rate (%)	Std. error	Weighted odds ratio (95% CI)	P-value
unweighted	personalized therapy	78	73. 1	0. 0521	1. 07 (0. 92, 1. 25)	0. 4
	Standard therapy	62	64. 5	0. 0585		
weighted	personalized therapy	78	74. 1	0. 0423	1. 2 (0. 71, 2. 07)	0. 5
	Standard therapy	62	70. 5	0. 0334		

**Figure 2:**
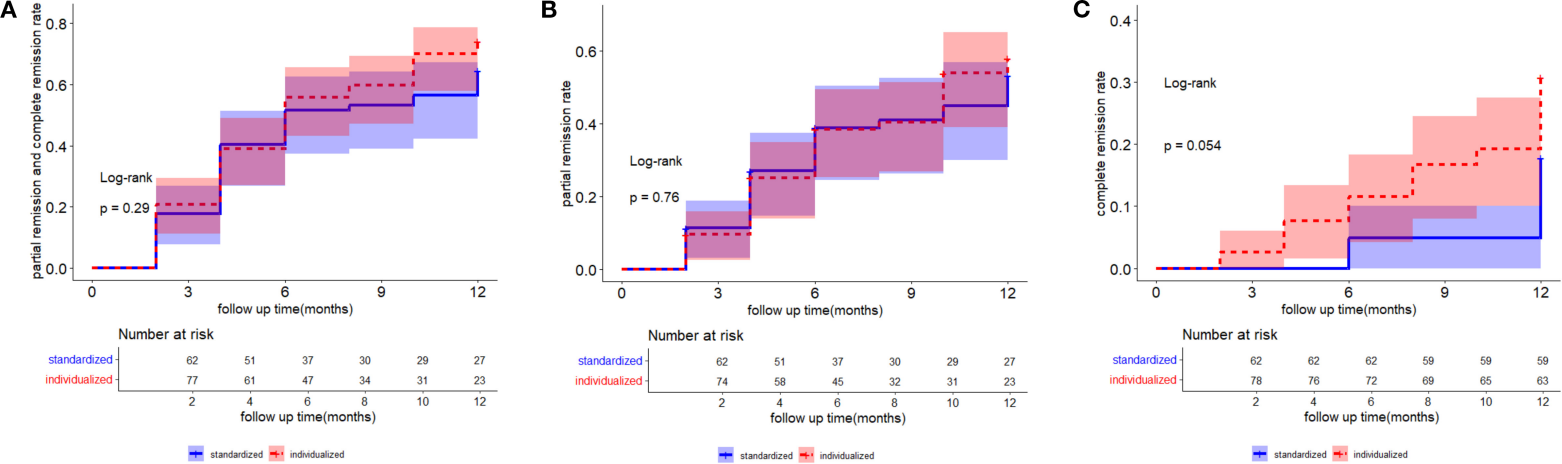
The log-rank test was used to compare the cumulative event rates of partial and complete remission **(A)**, partial remission **(B)**, and complete remission **(C)**. A two-tailed p <0. 05 was considered statistically significant.

The log-rank test was used to compare cumulative event rates of partial or complete remission (**Figure 2A**), partial remission (**Figure 2B**), and complete remission (**Figure 2C**). The results indicated that cumulative event rates between the two therapy groups were not significantly different.

### Secondary outcomes

From baseline to 12 months, the median urinary protein decreased from 7.33 g/d (IQR 4.73, 10.69) to 0.95 (IQR 0.23, 3.68) g/d in individualized therapy, and from 6.13 g/day (IQR 4.44, 9.54) to 1.36 g/day (IQR 0.40, 3.90) in standard therapy (**Table 2**, **Supplementary Figure 1A**, P = 0.445). Albumin increased from 22.90 (IQR 19.10, 26.70) g/L to 38.90 (IQR 34.30, 42.10) g/L in individualized therapy, and from 22.75 (IQR 18.15, 26.25) g/L to 38.30 (IQR 32.05, 41.05) g/L in standard therapy (**Table 2**, **Supplementary Figure 1B**, *P* = 0.957). Renal function remained stable over time in both groups (**Supplementary Figure 1E**).

Within the cohort of 110 participants available for anti-PLA2R antibody measurement at baseline, titers exceeded the threshold of 14 RU/mL used to define antibody positivity. Anti-PLA2R levels decreased in both groups during follow-up at a similar rate (**Supplementary Figure 1C**). Among 89 patients with detectable anti-PLA2R at baseline, 54 achieved anti-PLA2R negativity (<14 RU/mL) within 12 months after rituximab administration. Of these 54 patients, 47 subsequently achieved the study endpoint, compared with only 19 of the 35 patients without antibody negativity (*P <*0.001).

Most patients in the individualized therapy group achieved depletion of circulating CD19^+^ lymphocytes within 2 months after the first rituximab infusion, whereas all patients in the standard therapy group achieved depletion. The median time to CD19^+^ cell depletion was 2 months (IQR 2, 6) in individualized therapy and 2 months (IQR 2, 2) in standard therapy. CD19^+^ cell counts remained depleted in most patients in individualized therapy during follow-up, whereas recovery was observed from 6 months onward in the standard group, with significantly higher counts at 12 months [6.42 (IQR 2.24, 23.10) vs. 16.10 (IQR 5.23, 100.00), *P* = 0.024] (**Supplementary Figure 1D**).

### Rituximab regimen and cost

In individualized therapy, 78 patients underwent CD19^+^ and anti-PLA2R testing every 2 months. The median rituximab dose at 1 year was 800 mg (IQR 600, 1,100) per patient, with a total cost of RMB 16,227.5 (IQR 13,148, 23,536) per unit utility. In the standard therapy group, 32 patients received 1 g on days 0 and 15, and 30 patients received 375 mg/m^2^ weekly for 3 to 4 weeks. The median rituximab dose at 1 year was 2,000 mg (IQR 2,000, 2,400) per patient, with a total cost of RMB 31,265 (IQR 31,265, 40,163.2) per unit utility, which was significantly higher than that in individualized therapy (*P <*0.001). Detailed data are shown in **Table 2**.

### Relapse

Three patients in the individualized therapy group and two in the standard therapy group relapsed during follow-up. No statistical difference was found between groups (*P* = 0.814). One of the five patients who relapsed was antibody-negative, while the other four were antibody-positive. Of the five, two patients relapsed at the 1-year screening, probably due to re-emergence of anti-PLA2R autoantibodies at the 10-month visit. The other two relapsed without re-emergence of anti-PLA2R antibody or CD19^+^ B cells.

### Adverse events

Seven patients in the individualized therapy group and 10 in the standardized therapy group experienced at least one side effect, including infusion-related reactions and infections. No cases of leukopenia were observed. Two patients in the individualized group and four patients in the standard group suffered from pneumonia requiring hospitalization and intravenous antibiotics (see **Supplementary Table 1**, χ2 = 125.56, P<0.01). There were statistically significant differences in the frequency of adverse events between groups (χ2 = 89.167, P<0.01). No cancer diagnoses or deaths occurred during the trial (**Supplementary Table 1**).

## Discussion

In this prospective cohort study, we describe the outcomes of 78 IMN patients treated with a B cell- and anti-PLA2R antibody–driven regimen (individualized) compared with 62 IMN patients treated with standardized therapy, administered between 2019 and 2023, with follow-up for more than 12 months across 10 nephrology centers in eastern China. We found that the individualized rituximab strategy was noninferior to the standard rituximab strategy in inducing proteinuria remission at 12 months in patients with membranous nephropathy at moderate or high risk for progressive disease. The decline in proteinuria appeared more pronounced in the individualized group (73.1% of patients responded, with 34.6% achieving complete remission) than in the standard group (64.5% responded, with 17.7% achieving complete remission). Rituximab dosage has been widely reported, with multiple dosing strategies (8, 9, 17). As early as 2002, Remuzzi et al. in Italy reported satisfactory responses in eight refractory MN patients treated with standard therapy (375mg/mm^2^X4 doses) of the monoclonal antibody against the B lymphocyte surface antigen CD20-Rituximab (18). In more recent randomized controlled trials (RCTs), such as MENTOR (16), RI-CYCLO (19), and STARMEN (20), patients assigned to the rituximab group received 1,000 mg of intravenous medication on days 1 and 15 (16, 19, 20). However, similar remission rates were observed among the three treatment protocols for MN management (21). These protocols were: (i) 375 mg/m² weekly for 4 weeks (Regimen 1); (ii) 1 g on days 0 and 15 (Regimen 2); and (iii) 375 mg/m^2^ single dose followed by repeat dosing at 3–4 months (Regimen 3). The above RCT studies were the milestone of rituximab for the treatment of membranous nephropathy, however, in the MENTOR study, cyclosporine was discontinued after 1 year of usage in the control group. The RI-CYCLO study is currently the first RCT conducted to compare rituximab monotherapy with cyclophosphamide (CTX), however, cyclophosphamide was discontinued after half a year of use, thus the 2-year response rate might be overestimated in the RTX group (standard dosage) in MENTOR and RI-CYCLO studies. In addition, the STARMEN study is not a head-to-head study, RTX was used half a year later than cyclophosphamide, which can also contribute to bias. In our study, despite attempts at adjustment including IPTW, we cannot exclude the possibility of potential selection and confounding bias, especially the previous treatment. Confounding factor 1: The higher proportion of refractory IMN with previous immunosuppression treatment was a confounding factor which cannot be omitted. The subgroup analysis from the MENTOR trial (2019) demonstrated lower remission rates in refractory IMN patients in the RTX group. Data from the STARMEN trial (2021) suggested that refractory patients required longer treatment courses or combination therapy to achieve partial remission in the RTX group. Thus, a higher proportion of refractory IMN with previous immunosuppression treatment in the personalized RTX group might lead to a lower response rate than that in standard therapy. However, our study suggested a noninferiority response in the personalized group. Confounding factor 2: Patients in the standardized group had a higher level of education, received more family care, and had a greater awareness and understanding of the disease. However, the remission in personalized treatment was noninferior to the standard therapy group. Therefore, the influence of socioeconomic factors between the two groups may have underestimated the effectiveness of the personalized treatment. Confounding factor 3: There was a significant difference in renal function between the two groups. The personalized group had worse renal function than those in the standard treatment group (SMD = 0.639). Therefore, the influence of eGFR between the two groups might have underestimated the effectiveness of the personalized treatment.

Thus, the findings in our study may, to some extent, indicate a noninferiority response in the personalized group compared with the standard group. Further RCT studies are warranted to confirm our results.

Individualized administration has become an alternative for MN treatment. Furthermore, 1-year follow-up may preclude us from observing a more encouraging result. In many cases, patients with IMN can enter remission after 2 years, independently of the type of therapeutic strategies (19). The results also indicated that no significant difference was found in the 1-year recurrence rate between the two groups, perhaps due to insufficient observation time. Low-dose administration with continuous depletion of B cells has not yet shown its advantages; thus, a longer follow-up period is warranted to provide the answer.

There is a slight difference in the median (IQR) time to reach B cell depletion. A few patients did not achieve B cell depletion within 4 months in individualized treatment. However, due to the sustained B cell depletion achieved by subsequent administration, B cell numbers were still depleted in most of the patients in individualized therapy at one year follow-up. CD19^+^ B cells progressively recovered from 6 months in standard therapy, and the average B cell number was significantly higher than that in the individualized group at 1 year. Our results are in agreement with those of other studies, which found that low-dose, titrated B cell therapy in the state of continuous depletion can also achieve B cell depletion (19). Cravedi et al. compared the results of 12 patients who received a single dose of 375 mg/m^2^ with those of 24 matched patients who received a weekly dose of 375 mg/m^2^. At 12 months, the rate of nonresponders was 33% in both groups. Despite using the same dose of rituximab, Moroni’s results were less encouraging, with a lower response rate (8). One possible explanation for this difference is that Cravedi et al. administered an additional rituximab dose when more than 5 B cells/mm^3^ were detected in circulation. It has been indicated that persistent CD-19 depletion by rituximab is cost-effective in maintaining remission in calcineurin inhibitor–dependent podocytopathy (22). In our cohort, CD19^+^ B cell count was significantly depleted at the second injection two weeks after the first administration and remained depleted during follow-up in individualized therapy, which could provide evidence for the satisfactory results. Our individualized regimen is essentially a mini-dose, multiple-dose administration strategy, which can better achieve continuous B cell depletion.

At baseline, 78.5% of the patients were tested for anti-PLA2R antibody, of whom 80% were positive. Not all patients were tested for THSD7A-, NELL1-, or EXT1/2-related antibodies; otherwise, this would have affected the generalizability of the results (other antibody-related membranous nephropathy). It has been reported that disappearance of the phospholipase A2 receptor antibody is an early surrogate marker for clinical remission, confirming the predictive value of anti-PLA2R antibody negativity (23, 24). A rituximab protocol driven by anti-PLA2R was adopted and achieved a 91% response rate without side effects in 21 patients (25). Previous studies (16, 25) described MN patients with and without detectable anti-PLA2R and found that anti-PLA2R titer was marginally correlated with the amount of proteinuria. Consistently, a relationship was found between treatment response and anti-PLA2R titer at baseline. As expected, changes in anti-PLA2R predicted an increased probability of achieving the combined endpoint. Thus, individualized treatment is advantageous mainly due to the following factors. First, repeated or prolonged exposure to rituximab may induce antibody production, limiting the therapeutic effect or increasing the risk of immediate hypersensitivity after drug re-exposure. This may prevent patients who initially responded to rituximab from receiving a second course (26). Second, cumulative drug exposure was positively associated with risk. Fatal hepatitis B virus reactivation has occurred after rituximab monotherapy with standard four-dose regimens, as shown in studies using other lymphocyte-depleting agents such as Orthoclone OKT3 and thymoglobulin (27, 28). Therefore, titrating rituximab dosage according to circulating B cells and anti-PLA2R levels to minimize exposure may improve the safety of immunologic therapy.

The cost of rituximab also needs to be considered, especially in China. Because rituximab use is not covered by insurance in Chinese patients, the standard treatment regimen is extremely expensive for most patients with IMN (29, 30). Thus, physicians must consider patients’ financial burden and willingness to pay when providing medical advice (21). In our study, the total cost of individualized therapy was significantly lower than that of standardized therapy (*P <*0.001). Given the noninferior outcome between the two groups, individualized therapy can be more economical, safe, and personalized without diminishing efficacy. Furthermore, although the cost of rituximab is high, the total cost is relatively low owing to the long-term remission of IMN.

In this study, there were patients who did not respond to rituximab (RTX) treatment in both groups. Factors potentially influencing RTX efficacy may include reduced bioavailability of RTX due to urinary RTX excretion, anti-RTX antibody production, and chronic, irreversible damage to the glomerular filtration barrier (31, 32). Identifying RTX-sensitive patients prior to treatment remains a critical direction for future research. Furthermore, other techniques could also be considered to assess remission; for example, depletion of urinary podocytes might be associated with higher response in IMN patients (33).

This study has several strengths. First, the analysis included a large and recent cohort of patients with IMN from 10 sites in eastern China from 2019 to 2023. To our knowledge, this is the largest prospective cohort of IMN in China to date. Second, patients were closely monitored with predefined evaluations at each time point for statistical analyses. Logistic regression was conducted to optimize clinical equipoise between comparison groups, increasing the power of the analyses and the reliability of the findings. Furthermore, the targeted and personalized therapy conducted in Chinese IMN patients may provide useful information for future treatment decisions. Evidence of benefit from previous studies of rituximab in IMN supports the design of an adequately powered clinical trial to assess the benefit–risk profile of rituximab (11). Finally, reduced personal payment due to individualized therapy may help more than 70% of patients achieve remission and relieve their financial burden.

Our study is also subject to several limitations. It was not feasible to design a randomized controlled and blinded clinical trial, which could have led to imbalances in baseline characteristics and bias. To address this, we used logistic regression and applied IPTW to reduce potential bias. Second, most patients in the individualized therapy group had refractory membranous nephropathy and had experienced at least one course of immunosuppressant therapy (34, 35). Even 7.5% of the 140 patients had only received steroids, suggesting a lower possibility of remission even after switching to rituximab. Reassuringly, patients in individualized therapy showed a higher response rate than those in the standard group. It should be noted that after enrollment, tacrolimus and corticosteroids were gradually tapered and combined with rituximab. Since rituximab is not metabolized by CYP3A4, no data suggest rituximab has direct interactions with immunosuppressants such as corticosteroids or tacrolimus. Thus, the increased remission rate was largely due to the effect of rituximab. Another limitation is that only anti-PLA2R antibodies were tested at all participating sites. We cannot rule out the possibility of other novel antibodies in circulation among patients with undetectable anti-PLA2R (35–38). In addition, we only matched the types of drugs; the concentrations and treatment courses of immunosuppressants in the two groups were not further matched. Moreover, although steroid dosage was not more than 20 mg per day and immunosuppressant trough concentrations during observation were strictly controlled, we did not specifically calculate the different regimens between the groups, which is also a shortcoming of this article.

In conclusion, this multicenter cohort clinical trial indicated that individualized rituximab therapy was noninferior to standard therapy (1000mg X 2 or 375mg/m^2^ X 3~4) and may be a cost-effective and safe strategy for patients with membranous nephropathy. Prospective randomized trials are needed to investigate personalized treatment assignments as an alternative scheme for IMN and other autoimmune diseases (35, 39).

## Data Availability

The original contributions presented in the study are included in the article/**Supplementary Material**. Further inquiries can be directed to the corresponding author.

## Supplementary material

Supplementary Figure 1 Twenty-four-hour proteinuria **(A)**, serum albumin **(B)**, anti-PLA2R titer **(C)**, CD19^+^ B cell count **(D)**, and serial levels of eGFR **(E)** after rituximab treatment in individualized therapy (n = 78) and standard therapy (n = 62) patients followed up for 12 months.
