# Supplementary figures and images for: B cell and anti-PLA2R antibody-guided rituximab therapy in idiopathic membranous nephropathy: a prospective multi-center cohort study in the East Coastal Region of China
Source: Front Immunol. 2025 Sep 17;16:1633532. doi: 10.3389/fimmu.2025.1633532 (PMC12484018; doi:10.3389/fimmu.2025.1633532)

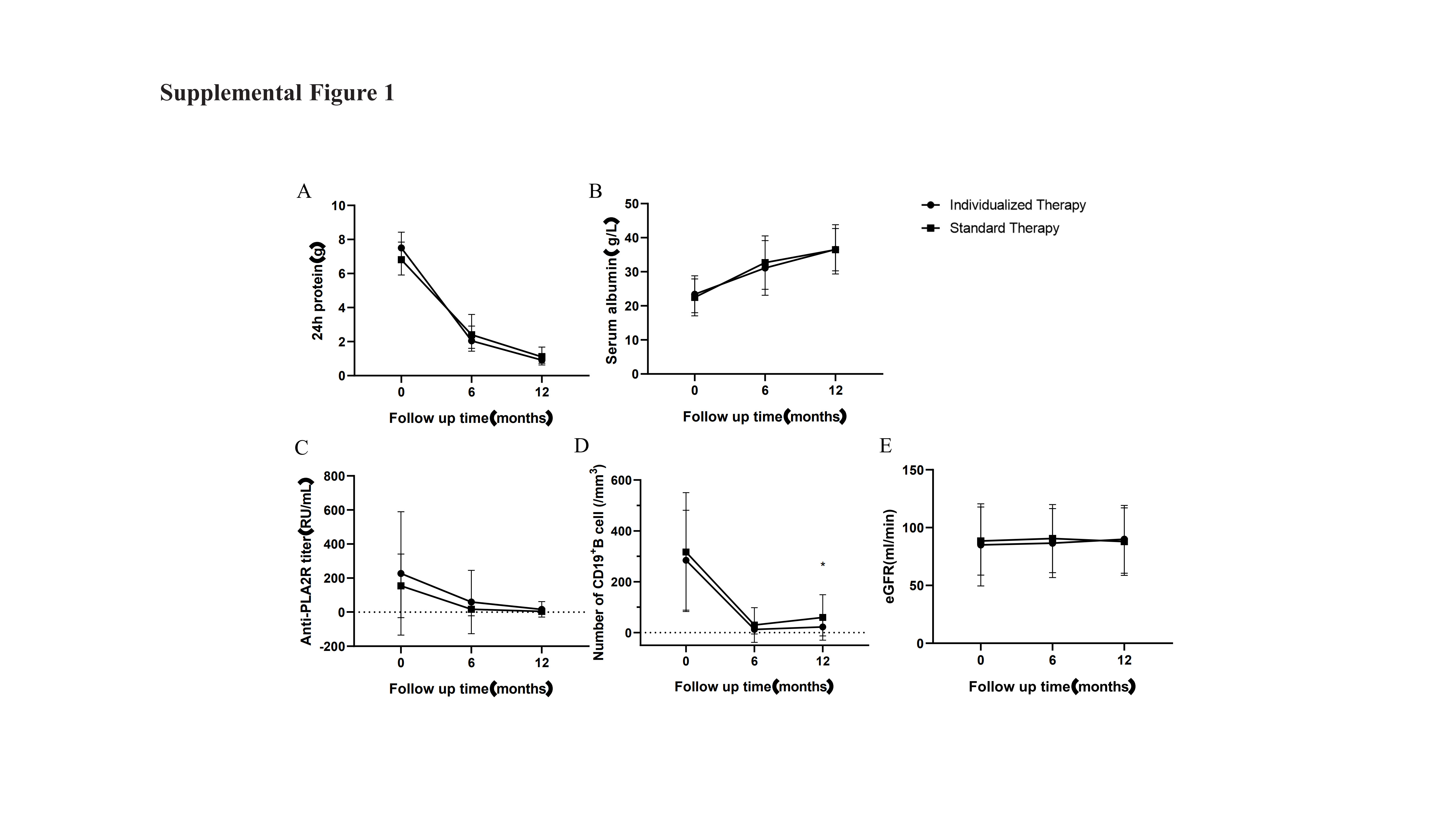

Supplement: Supplementary Figure 1 — Twenty-four-hour proteinuria (A), serum albumin (B), anti-PLA2R titer (C), CD19+ B cell count (D), and serial levels of eGFR (E) after rituximab treatment in individualized therapy (n = 78) and standard therapy (n = 62) patients followed up for 12 months. [file Image1.jpg]
