# Supplementary material for: B cell and anti-PLA2R antibody-guided rituximab therapy in idiopathic membranous nephropathy: a prospective multi-center cohort study in the East Coastal Region of China
Source: Front Immunol. 2025 Sep 17;16:1633532. doi: 10.3389/fimmu.2025.1633532 (PMC12484018; doi:10.3389/fimmu.2025.1633532)
Supplement: Supplementary file 2 [file Table1.docx]

**Supplemental Table 1**. Side effects events

| **Side effects events** | **Individual therapy**  **(n = 80)** | **Standard therapy**  **(n = 64)** |
| --- | --- | --- |
| n | 80 | 64 |
| Type of events |  |  |
| Infusion related reactions | 2 (excluded from statistical analysis) | 2 (excluded from statistical analysis) |
| Infection |  |  |
| Upper Respiratory tract | 1 | 1 |
| Pneumonia | 2 | 4 |
| Urinary tract | 1 | 1 |
| Skin | 1 | 2 |
| Digestive system |  |  |
| Diarrhea | 0 | 0 |
| Nausea | 0 | 0 |
| Major cardiovascular events | 0 | 0 |
| Mental nervous system: Headache, dizziness, abnormal sensation | 0 | 0 |
| Skeletal and muscular systems | 0 | 0 |
| Myelotoxicity |  |  |
| Anemia | 0 | 0 |
| Leukopenia | 0 | 0 |
| Thrombocytopenia | 0 | 0 |

Tips: it was a safety analysis set, thus two patients in each group suffered from infusion-related reactions were included.
